# Self, Me, or I? Unravelling the Triumvirate of Selfhood in Pathological Consciousness

**DOI:** 10.3390/brainsci15060640

**Published:** 2025-06-13

**Authors:** Alexander A. Fingelkurts, Andrew A. Fingelkurts

**Affiliations:** BM-Science—Brain and Mind Technologies Research Centre, Metsänneidonkuja 10, 02130 Espoo, Finland; andrew.fingelkurts@bm-science.com

**Keywords:** phenomenal consciousness, experiential selfhood, ‘self’–‘me’–‘I’, neurophenomenology, neuropsychopathology

## Abstract

In this conceptual review, we explore how alterations in the configuration and expression of the three core aspects of experiential Selfhood—‘Self,’ ‘Me’, and ‘I’—both reflect and shape an individual’s susceptibility to neuropsychopathology. Drawing on empirical neurophenomenological evidence and theoretical insights, we examine a range of psychiatric and neurological disorders through the lens of the Selfhood triumvirate. Our findings indicate that, despite variations in the expression of Selfhood aspects across different pathologies, their proportional configuration remains remarkably stable in most conditions, with the ‘Self’ aspect consistently dominant, followed by the ‘Me’ aspect, and finally the ‘I’ aspect. This stability suggests a fundamental neurophenomenological hierarchy in Selfhood organization, which seems to be disrupted only in extreme cases such as vegetative (unresponsive) states and also schizophrenia. Ultimately, we propose that all neuropsychopathologies are best understood as disorders of Selfhood, where disruptions in the dynamic balance and configuration of the ‘Self’, ‘Me’, and ‘I’ aspects accompany neurophenomenological manifestations in distinct dysfunctions and pathologies.

## 1. Introduction

For centuries, the mystery of consciousness has captivated philosophers and scholars alike. In recent times, scientists—particularly neuroscientists—have also joined the quest to unravel the enigma of consciousness. The past two decades have seen a surge of interest, with leading scientific journals dedicating Special Issues to various aspects of consciousness.

The scientific study of consciousness has traditionally focused on healthy participants across a range of experimental settings. In contrast, research examining consciousness under pathological conditions has been relatively isolated and lacking in systematic approaches. Yet, a structured and comprehensive investigation of altered states of consciousness across different pathologies could significantly deepen our understanding of the dynamics and boundaries of conscious experience.

Building on the abovementioned, in the present contribution, we will focus on various pathologies and their associated consciousness alterations.

The scientific exploration of consciousness has generated an extensive body of empirical and theoretical knowledge. However, this abundance of research has also led to a proliferation of definitions, terms, and classifications, often creating confusion. The field frequently encounters two logical fallacies: the *jingle fallacy*, where the same term is used to describe different phenomena, and the *jangle fallacy*, where different terms are applied to the same phenomenon [1].

To maintain clarity for the purpose of this review, we propose narrowing the scope of consciousness to its most *pathology-relevant aspects*, avoiding the introduction of new terms. First and foremost, it is important to distinguish consciousness from cognition—a conflation that remains common in both scientific and philosophical discussions [2,3,4]. While certain prominent neuroscientific theories, such as Global Neuronal Workspace (GNW) and attention schema theory, emphasize the role of access, attention, and metacognitive functions in conscious experience, they do not entail that consciousness is reducible to or dependent upon these cognitive processes [5]. Indeed, a broad body of neuroscientific, neuropsychological, and neurological evidence points to instances where consciousness and specialized cognitive functions such as memory, attention, language, introspection, or sensorimotor processing can dissociate (for reviews, see [6,7]). Moreover, interpretations inspired by integrated information theory (IIT) and historical views like that of Jaynes [8] suggest that consciousness may be grounded in structures or features that do not map neatly onto classical cognitive functions. Thus, while debates remain regarding the relationship between consciousness and cognition, we maintain that these constructs are conceptually and empirically distinguishable and should not be conflated (see also [3,4]).

Second, it appears that the mental/psychological effects of *any* pathology are deeply *personal*, and the same pathology may affect different people differently to some extent. *Subjective experience* is an extremely important aspect of consciousness: if a mental state is not subjective then it is not conscious [9]. Subjective experience itself is *phenomenal consciousness* [10,11,12,13]. We previously argued [14] that phenomenal consciousness encompasses all immediate and undeniable (from the first-person perspective) phenomena of subjective experiences that any person can have right here and right now. In this definition, phenomenal means subjective: someone has phenomenal consciousness if they are currently having any type of subjective experiences [15].

At the core of phenomenal consciousness lies the *self*, which ties together personal identity with multiple phenomenal experiences. This connection was recognized as early as 1794 by the German philosopher Fichte, who asserted that “[e]verything that occurs in consciousness is founded, given, and introduced by the conditions of self-consciousness” [16] (p. 50). In various forms, this idea has endured across philosophical and scientific traditions [17,18,19], where it has been articulated through frameworks such as the minimal self hypothesis and discussions of self-consciousness and mental ownership.

Among the various phenomenal objects (complex patterns of phenomenal qualities that are spatially extended and bounded together to form a unified Gestalt with a particular meaningful semantics that is immediately present for the person [10]) within phenomenal consciousness, the self holds a uniquely dual significance. On one hand, it appears as an ontological entity or distinct ‘object’ within subjective experience. On the other, it serves as the foundation for integrating representational content from a first-person perspective. As Humpston [20] argues, without a sense of self, awareness of any experience becomes impossible. Put differently, an ongoing experience only becomes conscious when a connection is made between the mental presentation of an event itself and the mental presentation of the self as the agent or experiencer of that event [21,22,23]. It is important to note here that some perspectives—such as the minimal self hypothesis—allow for the possibility that aspects of phenomenal experience may occur in the absence of a fully integrated or reflective sense of self [19] (see also [24]).

The importance of self becomes even more apparent when considering the following clinical evidence. We acknowledge that self is a multidimensional construct, encompassing various aspects such as the minimal self (immediate embodied self-experience), autobiographical self (linked to personal memory), self-concept (beliefs about oneself), and self-reflective awareness. The clinical evidence we present below spans these different dimensions, and although not referring to a single unified phenomenon, each case illustrates how self-related processing plays a foundational or resilient role in conscious experience and psychological functioning.

(i) Self-consciousness appears to be generally more resilient to brain damage than specialized cognitive functions [25]. For example, consider cases where self-consciousness was preserved despite (a) severe hydrocephaly, where massive ventricular enlargement left only a thin cortical mantle [26], (b) extensive brain damage, including the destruction of nearly one-third of the brain, encompassing the insula, anterior cingulate cortex, and medial prefrontal cortex [27], (c) widespread neural damage caused by herpes simplex encephalitis [28,29], (d) hemispherectomy, a radical surgical procedure removing an entire brain hemisphere, leaving the patient with the remaining hemisphere intact and functioning [30], and (e) ‘split-brain’ surgery [31,32,33].
(ii) In many cases, self-consciousness plays a critical role in enabling cognitive resilience and facilitating cognitive function recovery from impairments [25]. It appears that rebooting self-consciousness is often the first critical step in cognitive rehabilitation, preceding and enabling the recovery (along the regaining autonomy) of more specialized functions [7]. These observations are further supported by a condition called anosognosia, where individuals’ lack of self-awareness of their own deficits is negatively correlated with resilience [34].
(iii) Even in severe memory disorders, such as amnesia or Alzheimer’s disease, patients retain aspects of self-knowledge [23]. For example, despite losing access to their recent autobiographical memories, such patients can still describe aspects of their identity, such as their appearance, personality, and social relations, that are particularly important to their self-concepts [28,35,36,37,38].
(iv) Additionally, differences in self-perception influence how individuals interpret ambiguous situations, potentially contributing to the onset and persistence of emotional symptoms [39]. Furthermore, self-esteem (a component of self-concept) has a formative and sustaining function in individuals’ mental health [40,41].
(v) Moreover, self-consciousness alterations predominate the patient’s phenomenological experiences and may be either long-lasting or permanent [42]. See for example Kean’s own description [43] (p. 1034): “The clinical symptoms come and go, but this nothingness of the self is permanently there […] By nothingness, I mean a sense of emptiness, a painful void of existence that only I can feel. My thoughts, my emotions, and my actions, none of them belong to me anymore. This omnipotent and omnipresent emptiness has taken control of everything. I am an automaton, but nothing is working inside me.”
(vi) Finally, self-consciousness is foundational to an individual’s ability to have preferences regarding how their life goes and make meaningful choices about it [44].

Self enables the *first-person perspective* that is essential for experiential *Selfhood*. We previously proposed that the “experiential Selfhood refers to a sense of the undergoing experience in its implicit first-person mode of givenness that is immediately and tacitly given as mine […] and it is accompanied by a functionally autonomous experience of subjective confidence or certitude […], making it possible to be engaged in autobiographical thoughts involving semantic and episodic memory events related to self, as well as projecting the self into the future, thus enabling the sense of invariance of a narrative self over time […]” [45] (p. 182) (see also [46] (p. 23)). In this sense, Selfhood is the most central and private evidence of being an independent and free agent that unites embodiment, emotions, executive functions, attention, general intelligence, intention, and other components within the intra-subjective frame—first-person givenness [47].

The significance of the first-person perspective for experiential Selfhood is further supported by evidence from studies on dissociative identity disorder, in which patients experience separate ‘alters’ but typically remain aware of only one dominant alter at a time [28,48]. The full simultaneous embodiment of multiple alters is probably not possible. Indeed, “[…] at any one time exactly one world-for-me [is present] in which all my phenomenal experiences occur in a fundamentally interrelated fashion” [10] (p. xxi).

Selfhood is therefore not only central to understanding the *essence* of human experience but also to comprehending the moral significance of what makes life worth living [44,47,49,50]. Given this, our review will focus on the subjective experience of phenomenal self-consciousness within *experiential Selfhood*. However, because concepts such as selfhood, self-consciousness, phenomenal consciousness, and subjective experience are closely related yet distinct, some terminological clarification is in order. Self-consciousness refers to the capacity to represent oneself as the subject of experience—often associated with metacognitive or reflective processes. Phenomenal consciousness, by contrast, concerns the qualitative, subjective feel of experience (what it is like) independent of explicit self-representation. Selfhood encompasses both the structural and experiential dimensions of personal identity—such as ownership, agency, and temporal continuity (that give coherence to the self across time). Subjective experience serves as an umbrella term for any first-person conscious content, whether or not it involves explicit self-reference.

Investigating how self-consciousness manifests in various pathologies holds both clinical and theoretical significance. It can deepen our understanding of consciousness by revealing its phenomenological structure and potential underlying mechanisms. Studying alterations in Selfhood during pathological conditions where phenomenal content is altered may help us determine which aspects of self-consciousness are most tightly linked to disease and what are the relations between the brain’s functional properties and conscious subjective experiences.

## 2. Self-Consciousness in Sickness: A Methodological Remark

Clinicians are increasingly recognizing the importance of assessing the status of self-consciousness in psychoneuropathological conditions. This interest in alterations of self-experience has led to the term ‘self-disorders’ or ‘disorders of self’ [51]. “Self-disorders are non-psychotic, subjective anomalies. These disturbances affect the basic experiential self, which is at the root of existing as a self-present, demarcated and temporally stable subject of experience and action. This “core” or “minimal” self refers to the first-personal articulation of all experience (thoughts, images, perceptions etc.), also called “mineness,” “for-me-ness” or “ipseity” (ipse being Latin for self, itself) […].” [52] (p. 2).

However, the progress in the field of psychoneuropathology of self-experience has two key limiting factors:

(i) Most research on self-disorders has been limited to schizophrenia spectrum disorders [52,53,54] (just to mention a few). However, alterations in self-consciousness have also been observed in other conditions, for example, autism [55], cognitive disorders [56], vestibular disorders [57], and internalizing disorders (descriptive label uniting depression and anxiety disorders; see [58]).
(ii) Many studies treat self-consciousness as a singular, *unitary* phenomenon or equate it with only one of its aspects, such as the ‘minimal self’ or ‘narrative self’ [59], the ‘core self’ [28], the ‘pre-reflective self’ [60], or the ‘bodily-self’ [61]. However, using an oversimplified unitary or one-component conceptualization of self-consciousness makes it difficult to adequately describe/explain the variety of phenomenological manifestations within and between different neuropsychopathologies. A more productive approach conceptualizes self-consciousness as a dynamic, *multi-component* phenomenon, where self is a *complex pattern* emerging from the dynamic interactions of characteristic aspects, features, qualities, and components—none of which, on their own, are entirely sufficient or specific to define the self; only jointly do they constitute it [62]. Such a multi-component conceptualization of self-consciousness is more appropriate for capturing the rich multitude of phenomenological manifestations within and between different neuropsychopathologies. Additionally, it can achieve the following:
  - Explain cases where a person is aware of their unfolding experience yet lacks a sense of ego.
  - Differentiate between the experiencer (‘I’) and the object of awareness (‘Me’).
  - Capture the internal dialogue between the ‘I’ and ‘Me’.
  - Distinguish between the experience of ‘pure awareness’, ‘minimal phenomenal experience’, and ‘content-free’ awareness (along different features that characterize such states).
  - Provide insight into loss-of-self experiences, hyper-reflexivity, or an enhanced sense of self.
  - Recognize the phenomenological complexity of patients’ subjective realities.
  However, this pattern-based approach carries the risk of incorporating an open-ended list of aspects, features, qualities, and components which may arise ad hoc and ultimately diminish explanatory effectiveness. To prevent such vagueness, we propose that Selfhood can be meaningfully understood through a limited set of higher-order aspects that structure its complexity. Specifically, we identify three integrative aspects—phenomenal first-person agency, embodiment, and reflection/narration—each encompassing a range of more granular features. This structured, multi-component approach (as outlined in the following section) provides a balance between conceptual richness and analytical clarity.

In this context, we propose that alterations in self-experience should be considered the *clinical core* of *any* neuropsychopathology, as they are the only ones relevant to experiential Selfhood. Selfhood itself should be conceptualized as a multi-component construct in order to capture the wide range of phenomenological manifestations observed both within and across various neuropsychopathologies.

## 3. Experiential Selfhood: The Selfhood Triumvirate Model

The *Selfhood Triumvirate Model* builds upon the *neurophenomenological three-aspect construct* of the *complex experiential Selfhood*, initially proposed by Fingelkurts and Fingelkurts [63] and further developed with extensive empirical evidence [46,64,65]. This model is grounded in the following:

- *Neurophysiological evidence*: The existence of three major spatially distinct yet functionally interconnected brain subnetworks—also known as operational modules (OMs)—that together constitute the larger brain’s self-referential network (SRN) [46,63,64,66]. These OMs are estimated using quantitative electroencephalography (qEEG) as explained below.
- *Phenomenological distinctions*: The differentiation of three fundamental aspects of Selfhood, each associated with one of the three SRN’s OMs—phenomenal first-person agency, embodiment, and reflection/narration—all of which are commensurate with each other [62,67,68,69] and thus reflect the multi-faceted nature of self-consciousness [70].

**Figure 1:**
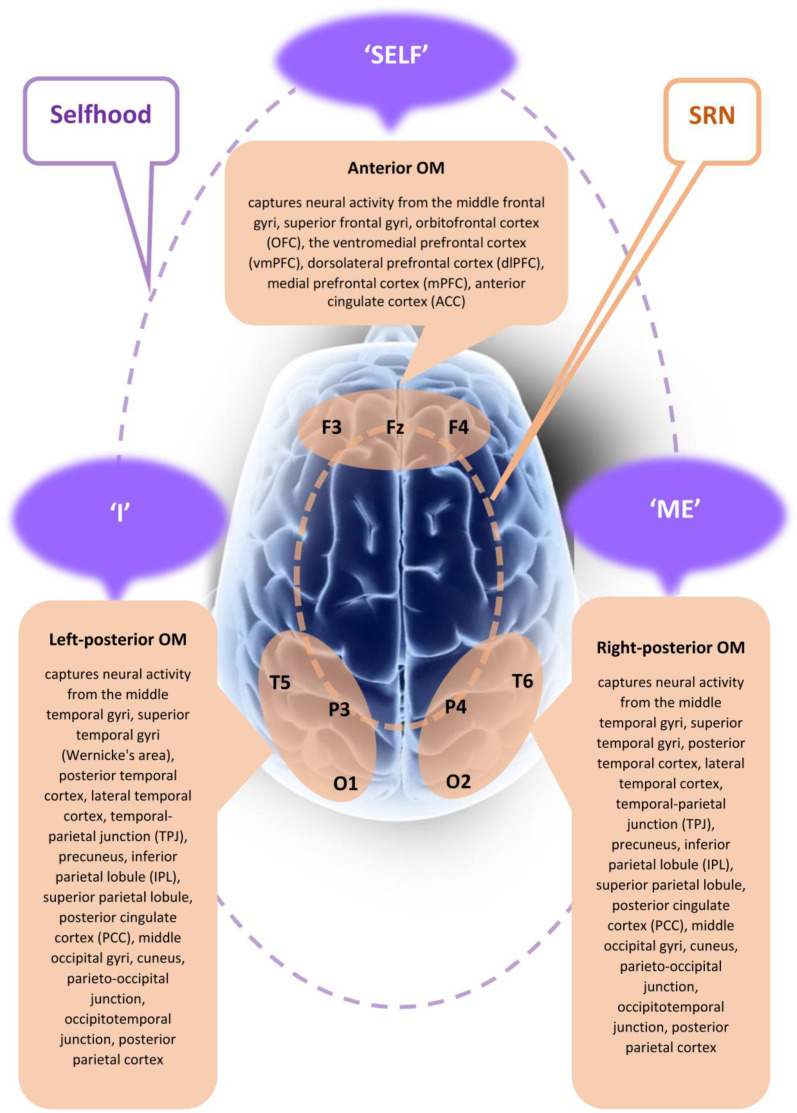
A graphical representation of the Selfhood Triumvirate Model. This figure illustrates three major spatially distinct yet functionally interconnected brain subnetworks, referred to as operational modules (OMs), that collectively form the self-referential network (SRN). Each OM integrates several local brain fields, as recorded by corresponding EEG electrodes. These local fields, in turn, represent synchronized activity of transient neuronal assemblies [14,15]. In the figure, OMs are depicted as orangish ovals, symbolizing operational synchrony across brain activities captured by EEG electrodes per every OM, and positioned on a schematic cortical map. The sets of brain regions included in the triumvirate model have previously been identified as integral components of the SRN [63]. Their inclusion was not arbitrary; rather, these areas consistently emerged as components of the three most stable, task-unrelated spatiotemporal patterns (OMs) which exhibited exceptionally high levels of operational synchrony in neurotypical individuals. Notably, this finding has been replicated in two independent studies conducted across two distinct ethnic-cultural populations and involving two different sensory modalities (see [63] for methodological details). Phenomenologically, the three OMs correspond to three fundamental aspects of Selfhood: ‘Self’ (first-person experiential presence or agency), ‘Me’ (embodied, interoceptive self), and ‘I’ (reflective, narrative self). Their dynamic interplay gives rise to a unified sense of Selfhood, visually indicated in the figure by the dashed purple circular line connecting ‘Self,’ ‘Me’, and ‘I.’ Abbreviations: EEG: electroencephalogram; OM: operational module; SRN: self-referential network; EEG electrode positions: F3—left frontal, Fz—frontal midline, F4—right frontal, T5—left temporal, P3—left parietal, O1—left occipital, T6—right temporal, P4—right parietal, and O2—right occipital. Clarifications regarding anatomical mappings: (a) the midline posterior cortex, a key SRN region [86], is represented here by the left and right precuneus (approximated by P3 and P4 electrodes), based on the spatial correspondences reported by Koessler et al. [87]; (b) while occipital regions (indexed by O1 and O2 and traditionally associated with visual processing) are not typically included as SRN components, numerous studies (e.g., [88,89,90]) have reported their involvement in SRN. These visual areas likely support the internal imagery crucial for self-related functions such as body representation, autobiographical memory, narrative construction, self-image, future planning, and reflective thought. Indeed, visual elements are consistently implicated in self-related processing [91]. The figure is adapted and conceptually inspired by Fingelkurts et al. [46].

According to the Selfhood Triumvirate Model, each of the three modules within the SRN corresponds to a distinct aspect/facet of self-consciousness (Figure 1):

The *anterior module* of the SRN is associated with the *phenomenal* first-person perspective and the phenomenal sense of agency, thus constituting *witnessing agency* or ‘***Self***’ in the narrowest sense (see [46] for a detailed description, supporting neural evidence, and empirical data), that is the phenomenal non-conceptual core in the act of knowing oneself [71], a sensed ‘centre of gravity’ [72] (see also [73]), where one has an experience of being directly and immediately present as the centre (or a focus) of a phenomenal multimodal perceptual reality [10,71,74,75,76]. “… [I]t is the Self that binds our fragmented representations of the world into unified, lived experiences. The link in a self-world coupling is therefore the Self itself” [77] (p. 161).

The *right posterior module* of the SRN is linked with the experience of self as a normally localized (via interoceptive and exteroceptive sensory processing) entity within bodily boundaries, as well *emotional states* and related *autobiographical emotional* memories, thus constituting *bodily representational–emotional agency* or ‘***Me***’ (see [46] for a detailed description, supporting neural evidence, and empirical data). Here, only a purely geometrical first-person perspective is present that originates from within the body representation, signifying an egocentric spatiotemporal self-model [71]. The body here is treated not as just another object of the physical world, but as a ‘vehicle’ that enables being a self in the world [78,79,80,81].

The *left posterior module* of the SRN is involved in the experience of thinking about and reflecting on oneself, including momentary narrative thoughts and inner speech, as well as reinterpretation of episodic and semantic memory events related to oneself, such as autobiographical storytelling, thus constituting *reflective/narrative agency* or ‘***I***’ (see [46] for a detailed description, supporting neural evidence, and empirical data). ‘I’ composes and edits the narrative of the information from ‘Me’ by creating “an internalized and evolving life story” [82] (p. 527) (see [83] for the ‘interpreter module’ as a seminal precursor to the narrative dimension). Thus, ‘I’ transforms ‘Me’ into “an internalized drama” [84] (p. 169) by constructing a dynamic autobiographical ‘worldview’—an introspectable narratively structured ‘landscape’ [2]. In a sense, ‘I’ constructs ‘Me’ as a reflexive conception [85].

Together, the three aspects (‘Self’, ‘Me’, and ‘I’) form a unified sense of experiential Selfhood [46,63]. In this triumvirate, the ‘Self’ observes, integrates, and unifies embodied self-awareness and emotional states (‘Me’) with narrative dimension and autobiographical structuring (‘I’).

Every aspect of the Selfhood triumvirate corresponds to a cluster of commonly occurring specific phenomenological experiences found in the general population across a wide range of conditions [65]. For instance, the ‘Me’ aspect encompasses experiences such as body image, body perception, body orientation and ownership, geometrical first-person perspective, physical agency, and emotional states; the ‘I’ aspect involves reflection, rumination, narration, autobiographical narration, and the structure (along the speed) of internal speech; the ‘Self’ aspect includes the phenomenal center, phenomenal first-person perspective, epistemic certitude, and the witnessing observer. Furthermore, the facets within each cluster (triumvirate aspect) have distinct relationship and functional weights that vary across individuals, contexts, and conditions. Because Selfhood is not static but *dynamic*, the proportions of the ‘Self’, ‘Me’, and ‘I’ aspects fluctuate within an individual over time and differ across people [69]. As such, experiential “[…] Selfhood, described in terms of brain SRN dynamics and related phenomenological descriptions, is not a fixed entity “living” on its own, but rather an ongoing emergent property generated by the dynamic interrelation of at least three brain SRN modules that support three phenomenological features (witnessing, self-reflection, and self-embodiment) of Selfhood, which are themselves also complex elements having their own composition. It seems that such a neurophysiological three-dimensional construct model of the complex experiential Selfhood matches well with phenomenological (as well psychological and also conceptual) distinctions between different aspects of self and treats them as commensurate to one another and not as opposites […]” [46] (p. 21). This complex and nested fluidity (with various weights of the ‘Self’, ‘Me’, and ‘I’ aspects) results in a multitude of conscious experiences, shaping both healthy cognitions and neuropsychopathological conditions [65].

Furthermore, these three aspects of the Selfhood triumvirate share several important characteristics. The following can be said about ‘Self’, ‘Me’, and ‘I’:

(i) Present (albeit asynchronously) since early childhood [92]*: Embodiment* (‘Me’) as a pre-reflective, non-conceptual, and pre-linguistic sense of one’s body begins to form the earliest, already in utero [93,94], and by the age of 2, infants can construct a body image of themselves as an entire object while also considering this image as a subject, i.e., as an active source of self-representation and with the capacity to internalize discrete emotions [85] long before they internalize cultural standards and knowledge. *Phenomenal point of view* (‘Self’), and thus first-person perspective, first starts to develop when a child is about 2 years old and is completed around the age of 4–5 years [95], allowing the child to represent him/herself not only as a core/center but also as a holistic entity in the act of knowing [71]—a prerequisite for awareness of the subjectivity of one’s own experiences. Finally, *narration* (‘I’) begins when the child becomes a fluent internalized language user, around the age of 4–5 years [96,97]; at that time, the concept of being a subject of experience is formed, allowing for deep self-reflection and identity formation.
(ii) Expressed along the entire continuum of functioning, from health to pathology [65].
(iii) Transdiagnostic [65], which means that changes in the ‘Self,’ ‘Me’, and ‘I’ are observed in multiple disorders across the spectrum of neuropsychopathology.
(iv) Reflected in the features of qEEG phenotypes. qEEG is a digitally recorded and algorithmically analyzed electrical activity generated by the brain. On one hand, qEEG reflects the brain’s inherent functional organization and dynamic activity structure, which are intra-individually stable traits, as demonstrated by test–retest reliability and genetic studies. On the other hand, because intrinsic brain activity shapes and conditions cognitive processes, information processing, self-regulation, decision-making, behavior, and consciousness, qEEG also serves as a reflection of neurophysiological predispositions underlying cognition, personality, temperament, and character. In this way, it captures an individual’s psychological and behavioral traits (for relevant references, see [98]). In this context, qEEG functions as an ‘interface’ between neural activity, personal experience, and behavior, making it a reliable indicator of neurocognitive efficiency and overall well-being. To measure three aspects of the experiential Selfhood (‘Self’, ‘Me’, and ‘I’), the qEEG operational synchrony analysis is used (see [99,100]). Operational synchrony in qEEG refers to a measure of the functional connectivity between different brain regions, based on the simultaneous temporal synchronization of local EEG signal segments (i.e., naturally existing quasi-stable microstates). It quantifies how often and how reliably these discrete EEG patterns occur in synchrony across spatially distinct electrodes (OMs), reflecting the coordination of functional neuronal assemblies underlying integrated cognitive or conscious processes [99,100]. Spatial resolution is often regarded as a limitation of qEEG. However, local qEEG is understood to represent a functional source—defined as the brain region(s) contributing to the activity recorded by a single sensor. A functional source is an operational concept that does not necessarily align with a distinct anatomical brain structure. It remains neutral regarding challenges related to primary source localization and volume conduction (for references, see [98]).

These characteristics of Self’, ‘Me’, and ‘I’ suggest that they are fundamental, primary aspects of phenomenal experience.

Importantly, a causal relationship has been found between these three phenomenological aspects/features of experiential Selfhood and the functional integrity of subnets of the brain’s SRN, as measured by the qEEG operational synchrony [46]. This opens up a practical possibility for evaluating neurophenomenological aspects of experiential Selfhood triumvirate by assessing their dynamics, proportions, and alterations during various neuropsychopathologies in relation to a normative healthy condition.

Before delving into the neuropsychopathology of the Selfhood triumvirate, we would like to discuss its intrapersonal variance. To properly assess the Selfhood triumvirate variability associated with neuropsychopathology, first, within-subject stability must be established. Because the Selfhood triumvirate is a neurophenomenological construct, it is natural that the proportions of ‘Self,’ ‘Me’, and ‘I’, their weights, and the strength of their interrelations vary in accordance with changes in phenomenology. However, these fluctuations are not random; they are most-likely personally specific, exhibiting trait-like properties [101] (see also [102,103,104,105]). Even though further research and formal studies are required to confirm this assertion, this statement is supported by the very high (up to 99%) within-subject stability and test–retest reliability of functional interrelations across cortical areas [106,107,108,109,110], including those implicated in ‘Self’, ‘Me’, and ‘I’ aspects of the Selfhood triumvirate [46,63]. Additionally, functional interrelations across cortical areas have also been reported to be heritable (up to 70%) [111,112,113,114], making them the heritable traits.

## 4. The Dynamics of the Experiential Selfhood Triumvirate in Neuropsychopathology

In a healthy, normotypical condition, the coherence of experiential Selfhood depends on a delicate balance between the expression of three interrelated aspects: ‘Self’, ‘Me’, and ‘I.’ It has been proposed that any given state of Selfhood is characterized by a distinct but typical *configuration* (*relative proportion*) and varying degrees in the *expression* of these three aspects, in accordance with the functional significance of the prevailing phenomenological manifestations of a particular experience. Furthermore, an individual’s liability or vulnerability to developing or manifesting a specific neurodysfunction appears to be linked to a specific configuration and/or expression of the ‘Self’, ‘Me’, and ‘I’ aspects [65], which are expected to exhibit *trait-like* properties [101,102,103,104,105].

From a *dimensional* perspective, the expression of individual experiential features exists along a continuous spectrum ranging from ‘healthy’ to ‘disordered,’ with extreme deviations from health potentially reflecting ‘abnormal’ or pathological states [115]. This perspective highlights the need to evaluate aspects of Selfhood in pathology not as a binary condition (healthy vs pathological), but as a continuum of configurations and expressions, ranging from optimal functioning to sub-optimal, inefficient, and dysfunctional.

**Figure 2:**
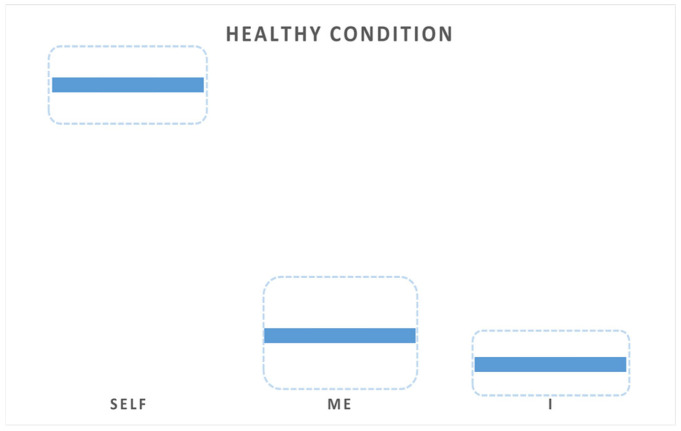
A graphical representation of the configuration (relative proportion) of the Selfhood ‘Self,’ ‘Me’, and ‘I’ aspects (solid horizontal bars) and their expression variability (dotted outlines), all of which are typical for resting healthy conditions. The y-axis is scaled to the maximum and minimum levels corresponding to the maximally and minimally expressed aspects of the Selfhood triumvirate, respectively. The specific values of expression are not important here, only their relative positioning within the Self–Me–I triumvirate. The manifestation of these three aspects of the Selfhood triumvirate was evaluated based on the functional integrity of three brain subnetworks within the self-referential network (SRN), assessed using qEEG operational synchrony measures [46,63].

In this context, a healthy, normotypical condition is characterized by a particular configuration (proportion) of the Selfhood ‘Self’, ‘Me’, and ‘I’ aspects and/or the magnitude of their expression and variability—all typically limited by a range that encompasses all phenomenological manifestations of healthy experiences [65]. In this healthy configuration, ‘Self’ is the most dominant aspect, followed by ‘Me’ and then ‘I.’ In terms of variability, ‘Me’ exhibits the greatest fluctuations, while ‘Self’ and ‘I’ vary relatively less (Figure 2).

This configuration of the Selfhood triumvirate—with ‘Self’ (related to anterior SRN module) as the most dominant aspect, followed by ‘Me’ (related to right posterior SRN module) and then ‘I’ (related to left posterior SRN module)—aligns with the findings of Thatcher et al. [106]. Their study, employing EEG coherence analysis, revealed that the functional integrity of frontal cortical regions was greater than that of posterior regions, and higher in the right hemisphere compared to the left. Notably, they also observed greater variance in the right parietal–occipital regions (corresponding to ‘Me’ aspect in Selfhood triad) than in the left (‘I’ aspect). This neurophysiological pattern was attributed by Thatcher et al. [106] to the differing contributions of long- and short-range association connections. Such structural–anatomical explanation can be enriched by a phenomenological perspective when considering the causal relationship between the three experiential aspects of Selfhood and the functional integrity of the brain’s SRN subnets, as measured by qEEG operational synchrony [46] (see also [63]).

In the normative state of consciousness, the ‘Self’ aspect (that correlates with functional integration of the anterior SRN module)—as the core witnessing presence and phenomenal center of gravity—constitutes the pre-reflective anchor of experience. It provides the stable phenomenological first-person perspective from which all experiential contents are apprehended. This explains its phenomenological dominance in the Selfhood triumvirate [46]. The ‘Me’ aspect, by contrast, represents the embodied and affectively grounded agency, subject to constant fluctuations in bodily states, emotions, and contextual salience. Its greater neurophysiological variability (as shown in EEG coherence and operational synchrony of the right posterior SRN module) mirrors its phenomenological dynamism: it is constantly shaped by interoceptive, exteroceptive, and emotional inputs [46]. Finally, the ‘I’ aspect—the narrative, linguistic, and autobiographical agency—operates with a more conceptual and symbolic modality. It is less dominant in moment-to-moment phenomenal salience, but provides temporal continuity and interpretive coherence. Its lower variability may reflect the more semantic and structured nature of autobiographical cognition, anchored in the left posterior SRN module associated with language and narrative construction [46].

Any deviation from this balanced ‘Self’–‘Me’–‘I’ configuration and/or in the magnitude of their expression and variability outside the healthy range on either side of the spectrum is expected to be associated with changes in the functionality of Selfhood. While transient shifts in Selfhood occur naturally in response to situational demands or intended for example during meditation, spontaneous and persistent alterations may signal an increased risk of neuropsychopathology.

Extreme deviations in the expression of ‘Self,’ ‘Me’, or ‘I’ aspects of Selfhood can lead to a fragmented or maladaptive self-experience, potentially contributing to neuropsychopathology (see below). Support for this came from a study where the authors demonstrated that both high and low values of self-concept lead to little or no sense of perspectival ownership of experience [116] or to dysfunction [117].

The neurophysiological underpinnings of ‘Self’, ‘Me’, and ‘I’ aspects of Selfhood are constrained by inherent biological limits as to how high or low the functional integrity of the SRN subnets associated with each aspect can be. Therefore, only the levels of optimal integrity (and thus expression of ‘Self’, ‘Me’, and ‘I’ aspects), associated with healthy conditions, provide sufficient ‘operating space’ for adaptability to regulate the balance of ‘Self’, ‘Me’, and ‘I’ based on internal and external demands within biologically feasible boundaries. Thus, one may conclude that the optimal level range is functionally maximally adaptive. Between optimality and dysfunction lies a broad spectrum of conditions associated with varying degrees of Selfhood triumvirate coherence and functionality. Adaptability here is understood in the sense of real-time functional regulation, not evolutionary fitness.

A dimensional approach to experiential features further suggests that (i) disruptions in Selfhood functionality may not progress in a linear fashion but may instead reach critical thresholds that mark transitions to more severe psychopathological states [115], and (ii) a particular configuration and expression of ‘Self’, ‘Me’, and ‘I’ aspects of Selfhood triumvirate may have a distinct meaning and reflect distinct underlying pathogenetic processes as a function of the overall context within which they emerge (‘contextual functionality’) [118].

The following sections explore how alterations in the Selfhood triumvirate manifest across different neuropsychopathological conditions.

The variability in the ‘Self’, ‘Me’, and ‘I’ aspects that make up a complex experiential Selfhood across a range of pathological conditions has been examined in a number of neurophenomenological studies [65]. The phenomenology was assessed by examining the mental structures of subjective self-experience through self-reports and replies to standardized questionnaires. The neurophysiological data were obtained through qEEG operational synchrony measurements of the functional integrity within three subnets of the brain’s SRN (as outlined above) that correspond to the three aspects of the Selfhood triumvirate [46,63].

Healthy individuals in a resting state with closed eyes, with no current or previous neurologic or mental complains, served as a healthy reference baseline for comparison with neuropsychopathological conditions. The eyes-closed resting-state condition was chosen deliberately due to its methodological and theoretical advantages: it minimizes external sensory input and maximizes spontaneous, self-referential mental activity (e.g., internal narrative, autobiographical memory, and top–down cognitive processing). This condition is particularly relevant for studies on Selfhood dynamics [46], as it provides a window into trait intrinsic neurocognitive processes (see [98] and references therein). Alterations of the ‘Self’, ‘Me’, and ‘I’ aspects of experiential Selfhood were expressed as a percent deviation from this baseline. Readers interested in methodological specifics and an in-depth discussion of each included study are encouraged to consult the original publications referenced below.

### 4.1. Depressed Selfhood

**Figure 3:**
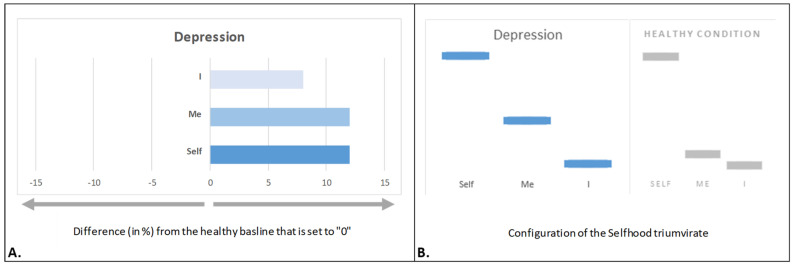
A graphical representation of percent deviation from the healthy baseline in the expression of the ‘Self’, ‘Me’, and ‘I’ aspects of the Selfhood triumvirate (**A**) as well as the relative configuration of these aspects within the Selfhood triumvirate (**B**) during depression. For (**A**): “0” indicates the healthy baseline (fully self-conscious healthy subjects at rest). For (**B**): the Selfhood triumvirate configuration for a healthy condition is presented for the reference. The manifestation of these three aspects was evaluated using a causally related neurophysiological proxy—qEEG operational synchrony measures of the functional integrity within subnets in the brain’s self-referential network [46,63].

In unmedicated outpatients diagnosed with major depressive disorder, the Selfhood triumvirate exhibits a pronounced overexpression of all three aspects, with the most significant increases observed in ‘Self’ and ‘Me’, followed by ‘I’ [119] (Figure 3A). In the context of this review, the ‘increase’/‘decrease’ refers to an ‘increase’/‘decrease’ in the intensity of a specific aspect of subjective experience (phenomenology) as well as an ‘increase’/‘decrease’ in activity and functional connectivity/integrity within a specific SRN subnet (neurophysiology). This pattern aligns with clinical observations of heightened self-focus, excessive rumination, and intensified embodiment in depression [28,120,121,122,123,124], constituting a *depressed Selfhood*.

It has been proposed that “these three components of complex Selfhood (indexed by distinct OMs of the self-referential brain network) synergize one another in a maladaptive loop and overtime become habitual, leading to a vicious circle that maintains a disordered affective state that clinically manifests as depression” [119] (p. 34). As a result, such depressive state gradually becomes part of the Selfhood.

The predominant position of increased witnessing agency (‘Self’ aspect) in depressed Selfhood (Figure 3A) suggests that the exaggerated self-focus is an important factor in the psychopathology of depression [122]. Because the phenomenal observing Self requires a ‘narrative interpreter’ in order to explain the exaggerated bodily sensations (‘Me’ aspect) and thus establish a complete self-awareness [125], the increased reflective/narrative agency (‘I’ aspect) explains increased maladaptive self-rumination during depression [126,127], which prevents depressed Selfhood from disengaging from negative thoughts and increased negative self-evaluation during depression. Furthermore, increased bodily representational–emotional agency (‘Me’ aspect) contributes to a well-documented increase in interoceptive awareness in depressed patients [124], leading to distorted body self-image [128] and negative emotional tone [129]. It appears that a simultaneous increase in all three aspects of the Selfhood triumvirate is a necessary pre-requisite for abnormal self-referential processes in patients with major depression [119].

Multiple regression analysis using one dependent factor (the Hamilton Depression Rating Scale—HAM) and three independent factors (expression of ‘Self’, ‘Me’, and ‘I’ aspects) as input parameters further supports this relationship: simultaneously increased expression of all three aspects of the Selfhood triumvirate is positively correlated with HAM scores, indicating that excessive self-referential processing is a key driver of depressive symptoms [119]. Thus, depressed Selfhood is trapped in a continuous positive feedback loop in which an excessive self-focus, exaggerated maladaptive self-reflection (rumination), and association of the self with negative emotions and body self-image reinforce each other [119].

Remarkably, although depression is associated with significant alterations in the expression of the three aspects, the overall configuration of the Selfhood aspects remains *relatively unchanged* (mirroring the pattern observed in healthy condition), apart from a slight increase in the relative separation between ‘Me’ and ‘I’ (Figure 3B). This suggests that while depression amplifies self-referential processes, it does not fundamentally alter the structural organization of Selfhood (proportional relation between ‘Self’, ‘Me’, and ‘I’ aspects). While we refer to major depressive disorder as a diagnostic label, we acknowledge that such categories are heterogeneous and may encompass distinct processes such as brooding rumination, which may be more directly linked to specific disruptions in self-related processing [130]. Our focus here is limited to observed configuration-level alterations in Selfhood structural dynamics within a representative clinical population with depressive symptoms.

### 4.2. Traumatized Selfhood

**Figure 4:**
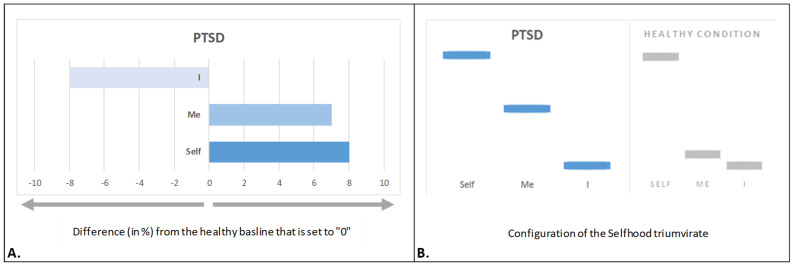
A graphical representation of percent deviation from the healthy baseline in the expression of the ‘Self,’ ‘Me’, and ‘I’ aspects of the Selfhood triumvirate (**A**) as well as the relative configuration of these aspects within the Selfhood triumvirate (**B**) during post-traumatic stress disorder (PTSD). For (**A**): “0” indicates the healthy baseline (fully self-conscious healthy subjects at rest). For (**B**): the Selfhood triumvirate configuration for a healthy condition is presented for the reference. The manifestation of these three aspects was evaluated using a causally related neurophysiological proxy—qEEG operational synchrony measures of the functional integrity within subnets in the brain’s self-referential network [46,63].

Selfhood alterations in individuals with post-traumatic stress disorder (PTSD) symptoms reveal a distinct pattern that constitutes a *traumatized Selfhood* [131]: heightened ‘Self’ and ‘Me’ expression, coupled with a reduction in ‘I’ (Figure 4A). This configuration aligns with clinical symptoms of hypervigilance, dissociation, and emotional reactivity in PTSD.

Excessive witnessing agency (‘Self’ aspect; Figure 4A) was associated with increased self-focus and hypervigilance/hyperawareness of the environment, often shifting one’s first-person perspective away from the present moment to the traumatic past (criterion E, DSM-5; [132]). Simultaneously, heightened bodily representational–emotional agency (‘Me’ aspect; Figure 4A) was significantly related to enhanced emotional, sensory, and bodily sensations (criterion D, DSM-5), such as fear, stress, and autonomic hyperarousal expressed as shivering, shaking, trembling, palpitations, and sweating [133,134,135].

Conversely, reduced reflective/narrative agency (‘I’ aspect; Figure 4A) was significantly associated with impaired linguistic processing and contextualization of traumatic memories, preventing their integration into a cohesive narrative (criterion C, DSM-5) [135,136].

Traumatized Selfhood (Figure 4A) suggests that constant hypervigilance coupled with profound emotional arousal would lead to sensory overload, but a lack of linguistic/contextual information and actual narrative would drive further exacerbated alienation of the self in PTSD [137]. According to Mucci, during trauma, Selfhood is unable to narrate the experience, which leads to an impaired capacity to remember; therefore, there is “no subject (just the body)” [138]. “The self is fragmented into a “me” and a “not-me” and any connection between the two has been severed. What the survivor manifests is painful state of concurrent awareness of a depleted self and of an intense experience that is disconnected and “forgotten”, but nevertheless affectively permeates and compromises life strategies of adaptation and defense.” [139] (p. 291).

Thus, it has been suggested that traumatized Selfhood “[…] is akin to ‘black hole’ that engulfs into itself every aspect of the self, resulting in substantial distortion to the overall sense of selfhood” [131] (p. 43).

However, despite significant alterations in Selfhood aspect expression, the structural configuration of the triumvirate remains *relatively unchanged*, mirroring the pattern observed in depression and healthy conditions, apart from a further increase in the relative separation between ‘Me’ and ‘I’ (Figure 4B). This suggests that PTSD, like depression, involves disruptions in self-referential processing rather than a fundamental restructuring of Selfhood (proportional relation between ‘Self’, ‘Me’, and ‘I’ aspects).

### 4.3. Depersonalized Selfhood

*Depersonalized Selfhood* is a defining feature of depersonalization disorder (DD), a condition characterized by a pervasive phenomenological sense of unreality and detachment from oneself, one’s own body, emotions, and autobiographical narrative. Individuals with DD often describe feeling like a lifeless robot or an automaton, observing their own bodily sensations, thoughts, and emotions from an external, detached perspective [140].

**Figure 5:**
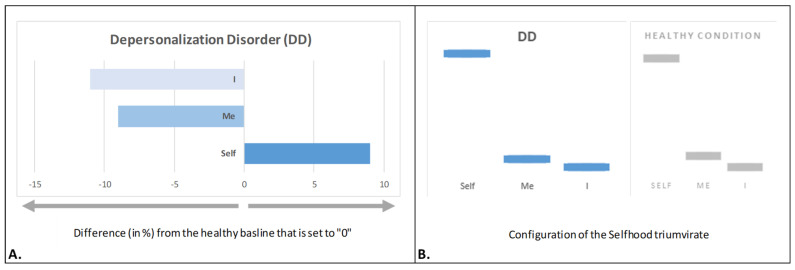
A graphical representation of percent deviation from the healthy baseline in the expression of the ‘Self’, ‘Me’, and ‘I’ aspects of the Selfhood triumvirate (**A**) as well as the relative configuration of these aspects within the Selfhood triumvirate (**B**) during DD. For (**A**): “0” indicates the healthy baseline (fully self-conscious healthy subjects at rest). For (**B**): the Selfhood triumvirate configuration for a healthy condition is presented for reference. The manifestation of these three aspects was evaluated using a causally related neurophysiological proxy—qEEG operational synchrony measures of the functional integrity within subnets in the brain’s self-referential network [46,63].

An analysis of the Selfhood triumvirate in DD [45] revealed a significant reduction in reflective/narrative agency (‘I’ aspect; Figure 5A), which was associated with difficulties constructing sequential and coherent autobiographical narratives—indicating impaired self-reflection. Additionally, a substantial decrease in bodily representational–emotional agency (‘Me’ aspect; Figure 5A) was associated with a distorted body experience, marked by a sense of disembodiment, diminished body ownership, impaired physical agency, and emotional numbness [45]. In contrast, witnessing agency (‘Self’ aspect) was significantly enhanced (Figure 5A) and was associated with an increased sense of detached observation of one’s own body, mental process, and life events [45]. It has been proposed that such hyper-observation or hyper-witnessing may serve as compensation for “[…] a profound lack of intentional reflection due to a loss of narrative flow and thus incapability to make sense (‘explain away’ […]) of the experienced disembodiment and lack of ‘mineness’, leading to even stronger feeling of alienation, being an automaton, a robot-like machine” [45] (pp. 196–197).

Furthermore, the severity of alterations in the Selfhood triumvirate aspects has been linked to the intensity of specific DD symptoms [45].

Remarkably, despite profound shifts in the expression of the ‘Self’, ‘Me’, and ‘I’ aspects, the proportional configuration of the Selfhood triumvirate remained unchanged in DD (Figure 5B), mirroring the pattern observed in the healthy condition and resembling those of depression and PTSD.

### 4.4. Psychotic Selfhood

**Figure 6:**
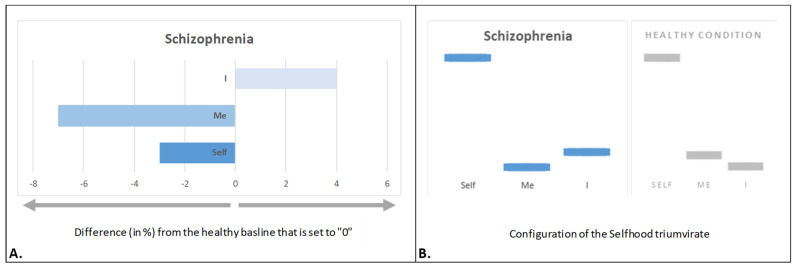
A graphical representation of percent deviation from the healthy baseline in the expression of the ‘Self,’ ‘Me’, and ‘I’ aspects of the Selfhood triumvirate (**A**) as well as the relative configuration of these aspects within the Selfhood triumvirate (**B**) during early schizophrenia in adolescents. For (**A**): “0” indicates the healthy baseline (fully self-conscious healthy subjects at rest). For (**B**): the Selfhood triumvirate configuration for a healthy condition is presented for reference. The manifestation of these three aspects was evaluated using a causally related neurophysiological proxy—qEEG operational synchrony measures of the functional integrity within subnets in the brain’s self-referential network [46,63].

In schizophrenia, Selfhood is profoundly disrupted, leading to dissolution of the “intentional arc” [141], as well as to experiences of detachment from both the body and the external world [142]. The study of early-stage schizophrenia in adolescents revealed a distinct altered pattern within the Selfhood triumvirate—*psychotic Selfhood* [65]: a marked reduction in bodily representational–emotional agency (‘Me’ aspect; Figure 6A), consistent with disturbances in bodily self-awareness, such as an unstable or fragmented body image. These disturbances contribute to a diminished sense of physical ownership, a loss of the feeling of mineness, and an impaired immediate, non-reflective sense of ‘me’ [143]. This was paralleled by diminished witnessing agency (‘Self’ aspect; Figure 6A), leading to a weakened sense of instantaneous self-identity [65]. Such loss of spontaneous self-awareness is poignantly illustrated in Kean’s personal account of schizophrenia: “Schizophrenia has silenced my real self, and even the observing self is biased by the process of subjective observation […] what scared me the most was a sense that I had lost myself, a constant feeling that my self no longer belonged to me” [43] (p. 1034).

These unusual experiences (lack of pre-givenness of mineness, agency, and bodily self-awareness reflected in diminished ‘Self’ and ‘Me’ aspects) force psychotic Selfhood to compensate by increasing reflective/narrative agency (‘I’ aspect; Figure 6A). This excessive introspection serves as an attempt to reconstruct a coherent sense of self in the absence of spontaneous self-experience [65]. The result is an intensified internal dialogue, often described as ‘thought pressure’ or a compulsive effort to rationalize and interpret one’s own fragmented self-awareness [143]. Sass [144] conceptualized this phenomenon as “hyperreflexivity”, wherein mental states are observed and analyzed rather than directly experienced. This relentless self-monitoring becomes a defining feature of psychotic Selfhood, as individuals attempt to make sense of their ontologically impossible experiences [20].

Indeed, “[w]hen a person is subjected to ontologically impossible experiences, their mind automatically begins a search for reason and meaning behind such experiences [‘I’ aspect] not only due to their bizarreness or invasiveness but also because they challenge and alter everything the mind takes for granted” [20] (p. 9).

Unlike the previously discussed conditions (depression, PTSD, and DD), early schizophrenia in adolescents was associated with not only substantial changes in the expression of the ‘Self’, ‘Me’, and ‘I’ aspects but also subtle alterations in the proportional configuration of the Selfhood triumvirate structure (Figure 6B). This suggests that schizophrenia may involve a more fundamental reorganization of self-experience, distinguishing it from other forms of psychopathological Selfhood.

### 4.5. Unconscious Selfhood

Studying unconscious Selfhood presents a significant challenge due to several key factors:

(i) Unconscious individuals are unresponsive and unable to describe/report their phenomenological experience;
(ii) When Selfhood is unconscious, it can be in either a *sub*conscious, *un*conscious, or even *non*conscious state—each with distinct implications for comprehending personhood and the moral significance of what makes life worth living (for a detailed description and analysis, see [47]).

A person may become unconscious due to a variety of conditions, including sleep (particularly NREM stage 3), anesthesia (e.g., with agents like propofol), syncope, absence seizures, or ‘disorders of consciousness’ (DoCs). For the purpose of this review, we will focus on DoCs, a broad term used in clinical practice to describe patients who have regained the ability to exhibit overt behaviors and are no longer comatose, but remain in a state of severely impaired awareness. These patients are typically in one of the following conditions: (i) vegetative state (VS) [145], recently renamed unresponsive wakefulness syndrome (UWS) [146], and defined as a “clinical condition of complete unawareness of the self and the environment” (Multi-Society Task Force on PVS [147] p. 1499), or (ii) minimally conscious state (MCS), which is a condition of severely altered consciousness in which minimal, fluctuating, and non-reflex behavioral evidence of self or environmental awareness is demonstrated [148].

The established causal link between the functional integrity of subnets in the brain’s SRN (measured by qEEG operational synchrony) and the associated ‘Self’, ‘Me’, and ‘I’ aspects of experiential Selfhood [46] provides an appealing possibility to assume at least some aspects of phenomenological experience (if present) in DoC patients who are otherwise unable to provide subjective accounts [50]. “This knowledge may give at least some hints as to whether the patient enjoys the moral status of the kind and degree that is sufficient for personhood (in other words be a subject of a life) or only to support some aspects of phenomenal self-experience, as for example, embodiment (pleasure and pain)” [50] (p. 4) associated with the ‘Me’ aspect of experiential Selfhood.

According to Levy [44], full moral status requires an individual to have an interest in life, a belief in their own continuity over time, and future-oriented desires that provide motivation to continue living—qualities likely dependent on the presence of both the ‘Self’ and ‘I’ aspects of Selfhood. This partially addresses the first challenge mentioned above.

To address the second challenge (see above), the distinction between the three phenomena (*non*consciousness, *un*consciousness, and *sub*consciousness) and their relations to the experiential Selfhood triumvirate have been previously provided [47].

In brief, *nonconsciousness* belongs to a *neurophysiological domain* (no subjectivity, [9]) that includes all core physical, biological, and neurophysiological processes in the brain, such as the neural and computational mechanisms, information acquisition and processing, and automatic decision-making. *Unconsciousness*, on the other hand, belongs to the *mental domain* and refers to mental processing that occurs entirely outside of consciousness (information is learned, stored, and recalled in an unconscious manner)—subjectivity without awareness [9]. Finally, *subconsciousness* is a subset of unconsciousness, in which mental processes with sufficient activation for conscious access are temporarily blocked from crossing a dynamic threshold of consciousness; therefore, they have the potential to burst into consciousness under certain conditions (see [47] for a detailed description and argumentation).

Studies indicate that in patients in persistent VS/UWS, the overall SRN functional integrity is reduced to stochastic or extremely low levels [149]. Applying the distinctions between *non*consciousness, *un*consciousness, and *sub*consciousness, this finding suggests that in permanent VS/UWS, Selfhood is entirely lost, leaving only *non*conscious, purely neurophysiological activity [47] (pp. 13–14). As a result, behavior in these patients is limited to rigid stimulus-responsive patterns akin to those observed in earlier phylogenetic animal lineages [150]. However, this does not preclude complex cognitive operations from occurring at a mechanistic level, enabling biological functioning and self-regulation [151,152] without phenomenological awareness [153,154].

Importantly, patients in VS/UWS who eventually regain consciousness—sometimes even years post injury—exhibit slightly higher than stochastic, yet still significantly impaired, levels of SRN functional integrity. This suggests an *un*conscious state of Selfhood that is “... a phenomenological state of selfless, bodiless and timeless presence, characterized by a lack of individual first-person perspective and an ‘emptying out’ of all phenomenological contents, including thoughts” [50] (p. 4).

By contrast, patients in MCS retain some degree of SRN functional integrity, allowing for an unstable or ‘flickering’ Selfhood—neither fully integrated nor completely fragmented. This fluctuating state of Selfhood is *sub*conscious [47] (p. 14) and is comparable to experience in dreaming [155] or being in altered states of consciousness [64].

**Figure 7:**
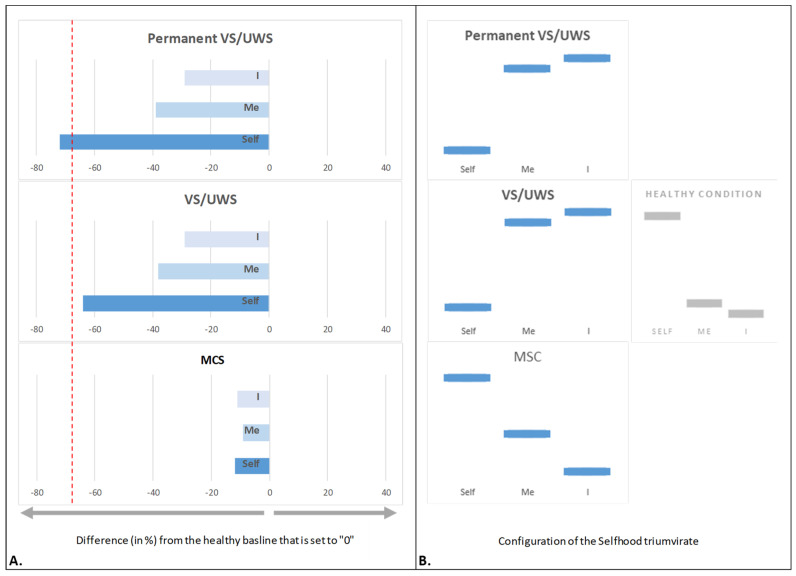
A graphical representation of percent deviation from the healthy baseline in the expression of the ‘Self’, ‘Me’, and ‘I’ aspects of the Selfhood triumvirate (**A**) as well as the relative configuration of these aspects within the Selfhood triumvirate (**B**) during permanent vegetative/unresponsive wakefulness syndrome (VS/UWS), VS/UWS, and a minimally conscious state (MCS). For (**A**): “0” indicates the healthy baseline (fully self-conscious healthy subjects at rest). The vertical red dashed line indicates the stochastic level of functional integrity. For (**B**): the Selfhood triumvirate configuration for a healthy condition is presented for reference. The manifestation of these three aspects was evaluated using a causally related neurophysiological proxy—qEEG operational synchrony measures of the functional integrity within subnets in the brain’s self-referential network [46,63].

When examining the three aspects of unconscious Selfhood in DoCs [156], the ‘Self’ aspect showed the most significant reduction among three aspects. Measured three months post injury, its decline reached stochastic levels in permanent VS/UWS, whereas VS/UWS patients who later (6 years post injury) regained consciousness showed a slightly less severe reduction (Figure 7A). In MCS, the ‘Self’ aspect was moderately diminished but not fully disrupted (Figure 7A). The fact that among three Selfhood aspects, only the ‘Self’ aspect showed important variability in its manifestation across different clinical outcomes suggests that its preservation plays a crucial (*unique*) role in the potential recovery of self-consciousness and the future survival of patients [19,156]. It seems that its resilience drives the recovery of other impaired Selfhood aspects and, as a result, many other cognitive functions [25].

Unlike other neuropsychopathological conditions reviewed above, VS/UWS is associated not only with a dramatic decline in all three aspects of Selfhood but also with a substantial reconfiguration of the Selfhood triumvirate structure (‘I’ > ‘Me’ > ‘Self’) when compared to a healthy condition (Figure 7B). This shift in the Selfhood configuration may reflect a rebalancing within the triumvirate, wherein the collapse of phenomenal self-presence and witnessing consciousness (the ‘Self’ aspect), together with a significant reduction in interoceptive and emotional embodiment (the ‘Me’ aspect), results in a state where the residual narrative ‘I’ persists without an experiencer or embodied grounding. In such a condition, the ‘I’ no longer functions as an active narrator but becomes a passive echo of previously encoded autobiographical or semantic content. It lacks the capacity to generate a real-time narrative anchored in first-person experience, bodily sensation, or emotional states that characterize the dynamic lived Selfhood experience.

Conversely, despite a noticeable reduction in the expression of all three aspects, the Selfhood triumvirate configuration in MCS remains similar to that of a healthy condition (Figure 7B).

In light of the previous findings regarding the causal relationships between the functional integrity of the three subnets of the brain’s SRN and their corresponding three phenomenological aspects of Selfhood [46], it appears that at least three different configurations of functional expressions of the ‘Self’, ‘Me’, and ‘I’ aspects of the triumvirate are associated with a lack of phenomenal sense of Selfhood, manifested for external observers as unconsciousness, but in reality, having a nuanced phenomenology, thus not being the unequivocal phenomenon (see Figure 4 in [47]):

(i) *Witnessing without content (experience of absence, nothingness, or emptiness)*: This state is characterized by the presence of normal or heightened ‘Self’ expression, with a simultaneous loss of both the ‘Me’ and ‘I’ aspects of the triumvirate. It is suggested [47] (pp. 14–15) that this configuration of the Selfhood triumvirate is associated with the experience of “being a phenomenal spatial-temporal (and often dimensionless) point, that observes and witnesses itself and the world” [64] (p. 264) due to the intact ‘Self’ aspect. This is accompanied by a total absence of content due to the loss of automatic and immediate sense of physical agency (the sense of disembodiment), as well as a decrease in the first-order experiential sense of ownership and emotionality [28,59,157,158] linked to a disintegrated ‘Me’ aspect and the lack of thinking, self-reflection, and personal narrative [159,160,161] associated with the disintegrated ‘I’ aspect of the triumvirate. Additionally, since it has been shown that the phenomenal sense of time arises from the embodiment sense maintained over time [162,163], one should expect a significant change in time perception (feeling of timelessness) when the sense of body is lost [64]. Despite being a ‘thin’ or ‘nonexplicit’ phenomenal experience [73,164], this ‘witnessing without content’ experience is nevertheless “... sufficient for creating a phenomenological centre of gravity and self-identification that is tied to an individual phenomenological first-personal givenness...” [64] (p. 266) (see also [165,166], and for recent empirical evidence, see [46,64]). Therefore, a patient in this state would maintain personhood with a distinct individual first-person perspective in accordance with Levy’s ‘full moral status’ postulate [49]. However, there would be a loss of awareness of being the same person extended over time. This is due to the absence of intact self-narration and autobiographical memory, typically supported by the ‘I’ aspect, which is disintegrated in this state. As such, based on Levy’s definition [49], this state only confers a partial moral status because it lacks the experience of a ‘life worth living’ [49,167].
(ii) *Observing agency is not present*: This state is distinguished by a significant loss of the ‘Self’ aspect of the Selfhood triumvirate, despite normal levels of the ‘Me’ and ‘I’ aspects. This Selfhood triumvirate configuration may imply [47] (p. 15) that there is phenomenal ‘emptiness’ or ‘nothingness’ because there is not anyone to whom the experience is occurring, not even the unextended point capable of epistemic self-identification [165,166]. Since the other two aspects of the triumvirate (‘Me’ and ‘I’) are functioning normally, there will be phenomenal states related to stimuli originating from both the outside and within the organism that are stored as memory traces; however, they will not be integrated within the first-person meaningful perspective [46]. In such a circumstance, when the sense of ‘Self’ has collapsed, binding fragmented representations of the internal and external world into unified, lived experiences is not possible. Reinterpreting Baars et al. [125], in this state, the phenomenal objects are not blocked from consciousness; rather, the observing subject is absent. Furthermore, in light of Levy’s ‘full moral status’ postulate [49], it is reasonable to expect that the patient will not have full moral status while being in this state. Although autobiographical memory events are phenomenally present, they are not present to any observing agent, as there is no witnessing entity capable of perceiving them from a phenomenal first-person perspective and to which the experiences are occurring [46,64].
(iii) *Complete dissolution of experiential Selfhood*: This state is characterized by a profound loss (complete disintegration) of all three aspects of the Selfhood triumvirate: ‘Self’, ‘Me’, and ‘I’. Such a state would represent [47] (p. 15) the total absence of all self-relevant phenomenological contents, characterized by “selfless, objectless, and timeless presence” [64] (p. 272), where the self-referential mechanisms for forming phenomenological events are suspended [166]. This state is generally marked by a significant lack of individual first-person perspective, a sense of witnessing agency, and ownership [46,64]. Additionally, subjective time (a sense of presence, past, or future) ceases to exist [46,64]. In this state, there is no phenomenality related to Selfhood, and therefore, patients in this condition lack a “locus of experience and self-ascription” [46] (p. 23), rendering moral considerations regarding personhood irrelevant [49].

Importantly, a six-year longitudinal study tracking self-consciousness recovery following severe traumatic brain injury (from MCS until full self-consciousness; 9 time points) found that the restoration of the ‘Self’, ‘Me’, and ‘I’ aspects of Selfhood triumvirate was (i) accompanied by progressive but stepwise improvement in functional integrity (measured by qEEG operational synchrony) in the three subnets of the brain’s SRN and (ii) correlated with clinical and behavioral improvement [168]. While the three aspects of the Selfhood triumvirate had the strongest correlations with their primary functions (as described above), the ‘Self’ aspect showed more global correlation. This is consistent with previous research and reinforcing the ‘Self’ aspect’s unique role among the three aspects [149,156] in the re-establishment of Selfhood. It has been proposed that the ‘Self’ aspect of the triumvirate serves as the foundation on which personal ‘autobiographical’, ‘narrative’, and ‘social’ selves (the ‘M’ and ‘I’ aspects) are built [156].

### 4.6. Other Pathological Conditions of Selfhood

The dynamics of the ‘Self’, ‘Me’, and ‘I’ aspects that comprise the Selfhood triumvirate have not yet been studied across all known pathological conditions (besides those described in the previous sections). However, the available literature implies that pathologies such as autism [55,169], bipolar disorder [170,171], cognitive disorders [56], anxiety [58], vestibular disorders [57], amnesia and Alzheimer’s dementia [28,35,36,37,38], Cotard syndrome (CS) [172], and others likely impact one or more aspects of the Selfhood triumvirate.

Among these conditions, Cotard syndrome stands out as particularly unusual and warrants a more detailed examination.

#### ‘Dead’ Selfhood

A compelling question arises: How can one possess a phenomenologically conscious Selfhood while simultaneously experiencing themselves as ‘dead’?

*‘Dead’ Selfhood* is typical for Cotard syndrome (CS), which is characterized by the phenomenological experience of being dead (nihilistic delusion): patients claim that they are devoid of bodily organs (including the brain), lack thoughts and emotions, do not have self, and do not exist at all [172]. ‘Dead’ Selfhood manifests through three primary distortions [173]: (i) *desomatization* (patients deny having real and lively bodily organs), (ii) *dementalization* (patients deny having memories, feelings, and sometime thoughts), and (iii) *death and nonexistence* (patients deny being alive and may even negate their very existence). Remarkably, patients with CS cease using the first-person pronoun ‘I’, instead referring to themselves in the *third person* [174]. This shift suggests that they can take themselves as an object of thought, signifying that the ability to form self-conscious thoughts remains intact [175].

This phenomenology of CS implies a breakdown in the balance of the Selfhood triumvirate. Specifically, one may expect that there is (i) a complete loss of the ‘Me’ aspect (desomatization and disembodiment), (ii) diminished expression of the ‘Self’ aspect (erosion of the phenomenal first-person perspective), and (iii) preserved or even heightened expression of the ‘I’ aspect (presence of self-reflective thought). This configuration of the triumvirate resembles the alterations seen in psychotic Selfhood (Section 4.4). Indeed, patients with nihilistic delusions typically suffer from schizophrenia [174]. However, while schizophrenia is marked by a diminished—but not entirely lost—‘Me’ aspect, CS probably involves a total absence of the ‘Me’ aspect. Without this ‘Me’ aspect, the ‘I’ aspect is left with no information to narrate, potentially leading to the illusion of nonexistence (being dead). Furthermore, the weakened ‘Self’ aspect reinforces this delusion by replacing the first-person perspective with the third person.

This hypothesis requires empirical validation, but it offers a compelling framework for understanding the nature of ‘dead’ Selfhood.

## 5. A Selfhood Triumvirate Neurophenomenological Perspective on Pathological Consciousness

The above review of various pathological conditions of Selfhood suggests that various psychoneuropathologies have distinct neurophenomenological profiles, where configuration and/or expression of the ‘Self’, ‘Me’, and ‘I’ aspects, measured neurophysiologically and phenomenologically, are affected differently [65]. Additionally, in all reviewed pathologies, the degree of alteration in these aspects correlates with the severity of specific symptoms (see references for the original studies above). Moreover, clinically significant functional recovery was associated with the return of the configuration and/or expression of these three aspects of experiential Selfhood to healthy condition functionality [168]. Functionality is understood here as the extent to which an individual is able to function, given varied degrees of manifestation of the phenomenological (mental) and associated neurophysiological (qEEG operational synchrony) characteristics [176,177].

Importantly, the reviewed empirical data suggest that there is no one set of Selfhood aspects for a healthy condition and another, distinct set for each pathological condition. Rather, (i) Selfhood is instantiated by a particular proportion and/or expression of the ‘Self’, ‘Me’, and ‘I’ aspects in the triumvirate in accordance with prevailing phenomenological manifestations of a given condition, and (ii) the ‘Self’, ‘Me’, and ‘I’ aspects of the Selfhood manifest along a unified continuum ranging from normal (healthy) to extreme (pathological) functioning. In this context, a given psychoneuropathology is characterized by (i) a concrete superposition of Selfhood aspects (configuration) that might differ significantly from a healthy condition and/or (ii) over- or underexpression of the ‘Self’, ‘Me’, and ‘I’ aspects of the Selfhood triumvirate (and the associated characteristics of qEEG phenotypes). Notice that, in comparison with a healthy state, deviations in the expression of the Selfhood aspects were evident (to varying degrees) across all reported neuropsychopathologies (see Section 4.1, Section 4.2, Section 4.3, Section 4.4 and Section 4.5). However, these deviations reached ‘true’ pathological levels—falling outside the variability range of healthy conditions—differently for different Selfhood aspects: (i) the ‘Self” aspect reached this level in all reported conditions, (ii) the ‘Me’ aspect did so in VS/UWS and depression, and (iii) the ‘I’ aspect did so in VS/UWS, MCS, and DD [65]. This means that not *all* alterations indicative of pathological Selfhood states are *necessarily* incompatible with normal functioning.

In this regard, pathological Selfhood can be conceptualized as an *adapted* state—a new *metastable* regimen of brain and mind functioning centered around altered homeostatic (neurophysiological) [178,179] and mental (phenomenological) levels. In this view, pathology gradually becomes part of the Selfhood. This adaptive process is known as allostasis, where a new stability is achieved through change, although it is already outside the normal homeostatic [180] and phenomenological ranges. In other words, the allostatic state is a state of chronic deviation in brain and mind activity from the normal state of operation with establishment of a new set point [181]. As a result, such Selfhood might be less able to cope with the demands of a constantly changing environment.

*Selfhood metastability* is defined here by the fact that the variability in the expression of the ‘Self’, ‘Me’, and ‘I’ aspects of the triumvirate is sufficiently large, and each of these aspects does its own ‘job’ while still retaining a tendency to be coordinated together to form a coherent experiential Selfhood. The interplay of these two tendencies (autonomy and integration) constitutes the metastable regime [182] of Selfhood functioning, where autonomous and integrated processes coexist as a complementary pair, not as conflicting principles [183]. From this perspective then, disorganization of the Selfhood can be viewed as a disorder of the metastable balance between the ‘Self’, ‘Me’, and ‘I’ aspects.

### The Resilience of Selfhood Configuration

Despite the altered expression of Selfhood aspects in various pathologies, the proportional configuration of the Selfhood triumvirate aspects remained remarkably stable in most conditions, with the ‘Self’ aspect as the constantly dominant component in the triumvirate, followed by the ‘Me’ aspect, and finally the ‘I’ aspect. Additionally, only the ‘Self’ aspect of Selfhood (i) was always pre-reflectively present in healthy subjects and most neuropsychopathological conditions, (ii) dominated the metastable imbalance between the ‘Self’, ‘Me’, and ‘I’ aspects for most neuropsychopathologies, (iii) either diminished considerably during MCS and VS/UWS or disintegrated during permanent VS/UWS, and (iv) predicted patients’ future survival [65].

Such resilience of the Selfhood configuration can be explained by a hierarchically organized neurofunctional constraint that reflects both evolutionary robustness and neurophysiological necessity. From an evolutionary perspective, the ‘Self’ aspect may constitute the most important component of *human* Selfhood, essential for survival [65]. It likely became instrumental in maintaining coherence across shifting internal and external demands, which in turn would have conferred adaptive advantages progressively acquired over time. The ‘Self’ aspect of the Selfhood triumvirate corresponds to the phenomenal non-conceptual core in the act of knowing itself [71,73]—the ability to phenomenally experience the world as ‘someone’ with basic perceptual, affective, and interoceptive coherence. It integrates bodily states, environmental stimuli, and fundamental affective valence, serving as the phenomenal foundation upon which the ‘Me’ and ‘I’ aspects are integrated within the wide spectrum of human behaviors and conditions [46]. As for the neurophysiological constraint, then pathologies that impair higher-order cognition (e.g., dissociative disorders, mild cognitive impairments) typically do not disrupt the fundamental capacity to have subjective experiences, maintaining the dominance of the ‘Self’ aspect [63]. Indeed, it has been demonstrated [65] that the ‘Self’ aspect has a wide range of expression in various pathological conditions while having a very narrow window for variance in a healthy condition. The ‘Me’ aspect is more vulnerable to disruption by different pathologies, though without erasing the subjective awareness itself, while the ‘I’ aspect, being the most abstract and reflective component of Selfhood, is also the most flexible and ‘dispensable’ in cases of severe dysfunction. This is so, because the ‘I’ aspect has a wide range of manifestations in a healthy condition and only a very narrow window for variation in ‘true’ pathological expression [65]. Only in cases of extreme disruption—such as vegetative states (where the ‘Self’ aspect collapses entirely) or schizophrenia (where the balance is altered by hyper-reflexivity and self-distortions)—does the whole Selfhood configuration undergo significant reorganization (see Section 4.4 and Section 4.5). This suggests that the Selfhood triumvirate follows a constrained model of neurophenomenological organization, where Selfhood remains anchored in a core proportionate structure of its three aspects (‘Self’, ‘Me’, and ‘I’), under most pathological conditions.

Taken together, the observations described above show that a significant decrease or even complete loss of embodiment and bodily originated geometrical perspectivalness (‘Me’ aspect) or narrative and conceptual self-reflection (‘I’ aspect) did not result in the disappearance of the most fundamental kind of phenomenal experience of being someone—a witnessing observer (‘Self’ aspect). Only a considerably diminished or entirely lost phenomenal sense of witnessing (‘Self’ aspect), when even the minimal phenomenal self as the abstract unextended point in space vanished, was associated with a significant decrease or complete absence of intentional content, phenomenal spatiotemporal self-location, and phenomenal first-person perspective (see also [46,64,65]). So, it appears that proper functional integrity of the ‘Self’ aspect of the triumvirate is a necessary and sufficient condition to support the basic feature of the complex Selfhood that ensures that one experiences oneself as a distinct epistemic entity located at the center of the phenomenal world one witnesses and observes.

## 6. Summary and Concluding Remarks

This review suggests that distinct neuropsychopathologies are manifested as disturbances in the metastable balance of dynamic configuration and/or expression of the ‘Self’, ‘Me’, and ‘I’ aspects that together constitute experiential Selfhood, in accordance with the functional relevance of prevailing phenomenological manifestations. The exact configuration and/or expression of these aspects vary depending on the phenomenology of a given pathological condition and correlate with specific symptomology. Furthermore, restoring balance within the Selfhood triumvirate appears to parallel clinically significant functional recovery, suggesting its potential role as a prognostic indicator for stable self-consciousness restoration.

In the context of empirical evidence and theoretical considerations presented in this review, we propose to rethink or reframe neuropsychopathologies as *disorders of experiential Selfhood*. Considering (i) a dimensional approach to neuropsychopathology (see above), (ii) the functional continuum of normality–pathology, recognizing that psychiatric and neurological disorders exist on a spectrum rather than as discrete categories (see above), and (iii) the fact that deviations in the expressions (though to varying degrees) of the ‘Self’, ‘Me’, and ‘I’ aspects of the Selfhood triumvirate are present across most psychiatric and neurological disorders due to their transdiagnostic nature (see above), neuropsychopathology can be conceptualized as (i) the degree of deviations in Selfhood aspects, assessed neurophenomenologically, and (ii) their disposition (stabilized relative to each other’s positions along the normality–pathology continuum), where the aspect with the largest deviation (or combination of several deviant aspects) may represent the leading pathogenic process or risk factor that manifests in the permanent mental constitution or as a heightened vulnerability to mental disorder. In such a way, the basis for neuropsychopathology may be a disruption in the metastable balance (configuration and/or expression) of the ‘Self’ ‘Me’, and ‘I’ aspects of experiential Selfhood.

Further, building on previous findings regarding the causal relationships between the three subnetworks of the brain’s SRN and their three corresponding phenomenological Selfhood aspects [46], we propose that the Selfhood triumvirate may (i) help in identifying whether and to what extent a patient possesses full or minimal self-consciousness, and whether specific aspects of Selfhood (or all three) are present, diminished, or absent, and (ii) distinguish between consciousness, UNconsciousness, NONconsciousness, and SUBconsciousness, offering insights into the potential (actual physical possibility) for Selfhood in unresponsive patients.

This knowledge has profound ethical implications, particularly regarding the moral status of patients. It may provide some hints as to whether an individual retains the degree of self-experience necessary for personhood—being a subject of life—or merely supports only some aspects of phenomenal self-experiences such as pain and pleasure.

To summarize, we argue that the Selfhood triumvirate framework offers a novel approach to understanding pathological consciousness, providing valuable insights into the neurophenomenological underpinnings of psychiatric and neurological disorders. Importantly, it highlights that normally adaptive processes may, under certain conditions, contribute to self-perpetuating pathological cycles that lead to various diseases within neuropsychopathology.

This framework has significant implications for (i) early identification and prevention by recognizing at-risk individuals before clinical symptoms fully develop, (ii) patient stratification by classifying individuals along the dimensions of ‘Self,’ ‘Me’, and ‘I’ expression for more precise diagnostic categorization, and (iii) personalized treatment strategies by tailoring interventions to an individual’s unique Selfhood configuration and expression of Selfhood aspects, potentially leading to more efficient and cost-effective therapeutic approaches.

## 7. Future Directions

This review suggests that alterations in the expediential Selfhood triumvirate should be considered a neurophenomenological core of all psychoneuropathologies. Framing pathological consciousness through the Selfhood triumvirate (‘Self’, ‘Me’, and ‘I’) opens several avenues for future research:

(i) To deepen our understanding of the variability and the variation limits of the Selfhood triumvirate aspects (‘Self’, ‘Me’, and ‘I’), a wider range of neuropsychopathological conditions should be examined using this neurophenomenological approach.
(ii) In order to effectively assess the variability in the Selfhood triumvirate associated with neuropsychopathological conditions, it is important to determine the trait-like properties of the ‘Self’, ‘Me’, and ‘I’ aspects. This can be achieved by investigating their within-subject stability and reliability in healthy populations.
(iii) To better grasp the lived experiences of patients—beyond classical symptomatology—neuropsychopathologies should be examined through the lens of the Selfhood triumvirate framework.
(iv) Understanding the distressing nature of altered states of Selfhood in neuropsychopathological conditions could benefit from comparisons with non-distressing altered states experienced during contemplative practices, such as meditation.
(v) Examining the discrepancy between the actual Selfhood and ideal Selfhood could enhance our understanding of the self-esteem construct. Evidence suggests that self-esteem has a causal role in the development of some mental health conditions [40] and that people with mental health conditions are found to experience a greater prevalence of discrepancies between the actual self and ideal self [41].
(vi) Further research is needed to identify thresholds in the variations in the ‘Self’, ‘Me’, and ‘I’ aspects of the Selfhood triumvirate that signal the transition from one functioning condition to another along the health–pathology continuum.

## Data Availability

Not applicable as this is a review article.

## Abbreviations

The following abbreviations are used in this manuscript:

CS	Cotard syndrome
DD	depersonalization disorder
DoC	disorder of consciousness
DSM	Diagnostic and Statistical Manual of Mental Disorders
GNW	Global Neuronal Workspace
HAM	Hamilton depression rating scale
MCS	minimally conscious state
OM	operational module
PTSD	post-traumatic stress disorder
qEEG	quantitative electroencephalography
SRN	self-referential network
UWS	unresponsive wakefulness syndrome
VS	vegetative state
